# Risk Factors for Sexual Offending in Self-Referred Men With Pedophilic Disorder: A Swedish Case-Control Study

**DOI:** 10.3389/fpsyg.2020.571775

**Published:** 2020-11-26

**Authors:** Felix Wittström, Niklas Långström, Valdemar Landgren, Christoffer Rahm

**Affiliations:** ^1^Center for Sexual Medicine (ANOVA), Karolinska University Hospital, Stockholm, Sweden; ^2^Department of Medical Epidemiology and Biostatistics, Karolinska Institutet, Stockholm, Sweden; ^3^Gillberg Neuropsychiatry Centre, Institute of Neuroscience and Physiology, University of Gothenburg, Stockholm, Sweden; ^4^Department of Clinical Neuroscience, Karolinska Institutet, Stockholm, Sweden

**Keywords:** pedophilic disorder, dynamic risk, child sexual abuse, case-control study, risk assessment

## Abstract

**Background:**

The risk of child sexual abuse among non-forensic, non-correctional patients with Pedophilic Disorder (PD) is largely unknown.

**Methods:**

We recruited a consecutive sample of 55 help-seeking, non-correctional adult men diagnosed with DSM-5 PD at a university-affiliated sexual medicine outpatient unit in Sweden. PD participants were compared with 57 age-matched, non-clinical control men on four literature-based dynamic risk domains and self-rated child sexual abuse risk.

**Results:**

PD participants scored higher than controls on all tested domains (0–3 points); expectedly so for pedophilic attraction (2.5 vs. 0.0, Cohen’s *d* = 2.40, 95% confidence interval (CI): [1.91–2.89]), but also for sexual preoccupation (1.6 vs. 1.0, *d* = 1.11, 95% CI: [0.71–1.51]), impaired self-regulation (1.4 vs. 1.0, *d* = 0.44, 95% CI: [0.06 to 0.81]), impaired cognitive empathy and antisocial traits (0.9 vs. 0.1, *d* = 1.18, 95% CI: [0.78–1.59]), and self-rated child sexual abuse risk (1.0 vs. 0.0, *d* = 1.56, 95% CI: [1.13–1.98]). When summarizing all five domains into a pre-specified composite score (0–15 points), PD subjects scored substantially higher than matched control men (7.5 vs. 2.1, *d* = 2.12, 95% CI: [1.65–2.59]). Five (9%) PD participants self-reported any previous conviction for a contact child sexual offense and eight (15%) for possession of child sexual abuse material or non-contact sexual offending (adult or child victim). Eighteen subjects (34%) acknowledged past week, child-related sexual behaviors.

**Conclusion:**

Self-referred, help-seeking men with PD scored higher (small to very large effect sizes) than non-clinical control men on psychiatric measures of dynamic risk of child sexual abuse suggested in prior research with correctional samples diagnosed with PD. Our findings, including the composite risk measure, might inform clinical practice, but needs validation against actual sexual offending behavior.

## Introduction

Pedophilic Disorder (PD) is defined as intense and persistent sexual attraction to prepubescent children associated with negative consequences for the individual or others (American Psychiatric Association, 2013). Although many individuals with PD do not sexually abuse children (Martijn et al., 2020), PD remains an important risk factor and treatment target in child sexual abuse prevention (Jordan et al., 2011; Association for the Treatment of Sexual Abusers [ATSA], 2014; Baur et al., 2016) and an estimated 50% of convicted child sexual offenders may meet PD diagnostic criteria (Seto, 2018).

A methodological problem with prior PD research is that most studies concern individuals involved with the criminal justice system. This entails substantial risks that findings secondary to selection biases following subject status as an identified and convicted child sexual offender diagnosed with PD may be over-interpreted as causal for the pedophilic attraction as such.

Individuals are increasingly seeking help for PD in sexual medicine contexts and general psychiatry settings, such as the German prevention project Dunkelfeld (Schaefer et al., 2010; Knack et al., 2019). Treatment decisions are partly based on clinically perceived risk of child sexual abuse, and current guidelines suggest SSRI medication or psychotherapy for low risk and antiandrogen medication for high risk individuals (Thibaut et al., 2010). However, validated measures of reoffending risk such as the Static-99R and STABLE-2007 (Hanson et al., 2016; Brankley et al., 2019) were developed in correctional settings and have limited utility with non-offender, at-risk individuals. Hence, risk assessment tools, addressing particularly dynamic, potentially changeable risk factors, are needed also for general psychiatry and sexual medicine. Improved knowledge about sexual offending risk among self-referred individuals with PD could advance treatment tailoring and the prevention of child sexual offending (Laws, 2000; Långström et al., 2013; Khan et al., 2015; Knack et al., 2019).

Assessing risk of sexual offending is important but complex and ethically challenging (Craig et al., 2005; Khan et al., 2015). Dynamic and static recidivism risk factors, although not necessarily causal, have indeed been identified among *convicted sexual offenders*. A classic systematic review (Hanson and Morton-Bourgon, 2005) found deviant sexual interest (e.g., pedophilia), sexual preoccupation, impaired self-regulation and antisocial traits among the most prominent dynamic or potentially changeable risk factors (Cohen’s *d* = 0.2–0.4) for sexual offense recidivism in known sexual offenders. Further, cognitive empathy has been linked theoretically to antisocial behavior and a recent meta-analysis suggested that it is moderately lower (Hedges’ *g* = −0.58) among child sexual offenders compared to the general population, but not compared to sexual offenders of adults (Morrow, 2019). The *motivation-facilitation* model for sexual offending (Seto, 2019) suggests that pedophilic interest and hypersexuality are *motivational* factors, antisocial behavior *trait factors*, and impaired self-control a *state factor*. However, sexual offending risk and actual offending behavior is mostly unknown among individuals *who voluntarily seek treatment for PD* without prior identified child sexual offending (Schaefer et al., 2010; Dombert et al., 2016).

We assessed the prevalence of dynamic risk factors for sexual offending in self-referred, non-correctional adult men diagnosed with PD and age-matched control men. We also asked for self-reported sexual offending behaviors against minors. Finally, to synthesize the empirical and theoretical literature, we constructed a pre-specified composite risk score. We hypothesized that PD participants would score substantially higher than controls on all five risk domains and the composite score.

## Methods

### Setting

This case-control study was part of the research project Pedophilia at Risk—Investigations of Treatment and Biomarkers (PRIOTAB), conducted from March 2016 to April 2019 at the Karolinska University Hospital in Stockholm, Sweden. PRIOTAB included clinical interviews, psychological testing and self-reports, neuroimaging, blood sampling and enrollment in a double-blind, randomized controlled efficacy trial (RCT) of the testosterone-suppressing medication degarelix (Landgren et al., 2020). Individuals calling PrevenTell^1^, a national telephone helpline addressing *unwanted sexuality* were screened for PD and invited to participate. PrevenTell is a low-threshold service providing counseling and treatment for sexual risk behaviors. The target group is people with self-experienced risky behavior, compulsive preoccupation with sexual thoughts or actions, sexual interest in children or impulses to force someone into sex. PrevenTell has been providing services since 2011, financed through the tax-funded health care system and targeted support from the Swedish Government. PrevenTell has received public visibility through repeated media coverage and increasing exposure in online search engines, and receives an average of four calls per day.

### Participants

**FIGURE 1:**
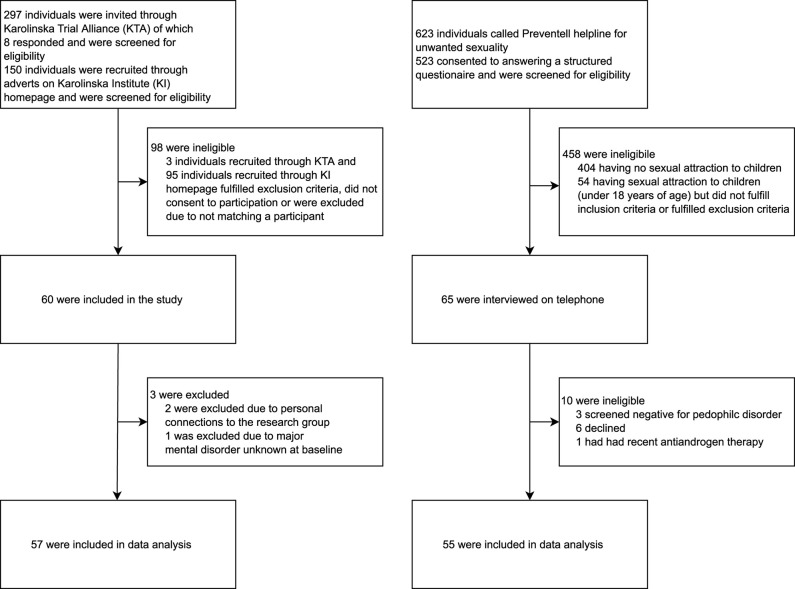
Flow chart for study recruitment of matched population control men and help-seeking, non-correctional adult men with Pedophilic Disorder in Sweden.

Men aged 18–66 years with DSM-5 PD ascertained by telephone interview prior to intake and confirmed on site by a board-certified general psychiatrist were eligible for study inclusion. Psychiatric exclusion criteria included severe psychosis, severe and acutely increased substance misuse, or suicide risk, respectively. Medical exclusion criteria included contraindications to magnetic resonance imaging (MRI) and drug trial inclusion; previously known or newly ascertained severe osteoporosis, electrocardiogram-verified prolonged QT/QT interval (> 450 ms), kidney or liver insufficiency, severe asthma, hypersensitivity to study drug or participation in another drug study during the previous 3 months. Karolinska Trial Alliance assists Karolinska University Hospital in recruiting participants for clinical trials and, along with independent study monitoring, helped the PRIOTAB project by disseminating information about the opportunity to participate as a non-patient control in the study. Healthy controls were recruited primarily through adverts on Karolinska Institutet’s homepage, and through Karolinska Trial Alliance. Exclusion criteria were the same for PD participants and controls. Finally, 55 PD participants and 57 age-matched controls were included in data analyses (Figure 1).

### Procedure

PD men and age-matched control men were all examined by a psychiatrist, completed self-rating questionnaires, provided blood samples, and underwent neuropsychological testing by a clinical psychologist. Self-report measures were filled out in privacy on site and participants were instructed to ask study staff for assistance if they had questions or difficulty answering the questionnaires. All participants underwent assessments during the same time of the day starting in the morning. Approximately 6 h were required for the assessments, the first two provided data presented in this case-control study.

### Ethical Considerations

A designated research nurse oversaw procedures where patient identification was necessary for clinical safety and insurance reasons; research subjects were otherwise only known to researchers by their initials. Participants were initially informed about health professionals’ obligation according to the Swedish Social Services Act to immediately notify the social services when a named child is at imminent risk of abuse or maltreatment. Subjects were asked at every visit to inform about such children in their vicinity. According to Swedish law, professionals are also allowed, but not obliged, to supersede confidentiality and tell the police any potential crime against children admitted by a patient. If a study participant reported any such actions, the choice to report or not to the police was discussed with an external ethico-legal advisory board linked to the Karolinska University Hospital. The study was approved by the Swedish Central Ethical Review Board (no: Ö 26-2014). Participants were offered reimbursement for transports to study visits and a financial compensation of SEK 1000 (the equivalent of 94 Euros before taxation) upon study completion. All subjects provided oral and signed informed consent and were offered treatment as usual after the study.

### Instruments

We compared PD and control participants on three self-reports, the Sexual Desire Inventory (SDI) (Spector et al., 1996) measuring sexual interest; the Hypersexual Behavior Inventory (HBI) (Reid et al., 2011) assessing hypersexuality; and the Ritvo Autism and Asperger Diagnostic Scale—Screening Tool (RAADS-14) (Eriksson et al., 2013) mentalizing subscale. We complemented this with three expert-administered measures; Conners’ Continuous Performance Test—2nd edition (CCPT-II) (Conners, 2000) tapping impulsivity; the Reading the Mind in the Eyes Test (RMET) (Baron-Cohen et al., 2001) addressing the understanding of others’ emotions; and Antisocial Personality Disorder (ASPD) symptoms according to the MINI Neuropsychiatric Interview 6.0 (Sheehan et al., 1998).

Additionally, participants completed measures addressing pedophilic attraction and related behavior and self-rated risk. We evaluated PD symptoms based on DSM-5 criteria and used a prespecified composite measure (SChiMRA, see Supplementary Material) developed by us to quantify self-rated risk of sexual offending against children and past week sexually abusive behavior toward children.

The five putative dynamic risk domains for child sexual abuse; pedophilic attraction (DSM-5 diagnostic criteria), sexual preoccupation (SDI, HBI and ongoing offensive behavior according to SChiMRA-B), impaired self-regulation (CCPT-II), impaired cognitive empathy and antisocial traits (RAADS-14, RMET and ASPD symptoms according to MINI 6.0), and self-rated child sexual abuse risk (SChiMRA-A), were assessed and compared between PD participants and control men. We predefined the composite score as described in Supplementary Table 1 by assigning 0–3 points to each of the five domains and summing them with equal weights to a total score ranging from 0 to 15. Hence, the composite risk score is based on self-, expert-completed and behavioral measures and potentially treatment sensitive since all five included domains could capture change over time. The five-domain score was used as the primary outcome measure in the PRIOTAB RCT (Landgren et al., 2020). Here we report both the five-domain score and a four-domain score (range 0–12) excluding pedophilic attraction; part of the inclusion criteria for PRIOTAB but also the grouping criterion for the present case-control study. A detailed description of the instruments is provided in Supplementary Material.

### Statistical Analyses

We computed Cohen’s *d*s with 95% confidence intervals to express effect sizes of potential differences between cases and controls. Using the freely available Practical Meta-Analysis Effect Size Calculator (Effect Size Calculator, 2020) provided by the Campbell Collaboration and based on Lipsey and Wilson (2001), Cohen’s *d*s were calculated from 2 by 2 (occasionally 3 by 2) tables for binary (frequency) data, from standardized mean differences for parametric test results and based on *p*-values and sample sizes for non-parametric test results. Following Cohen (Cohen, 1988), Cohen’s *d*s were interpreted as small (0.20–0.49), medium (0.50–0.79) or large (0.80+) effects.

Some data are missing since study participants occasionally did not complete self-rating questionnaires and no imputation procedures were employed. The number of individuals with complete data for each variable is reported in table footnotes. Data were analyzed using SPSS Version 24 for Windows.

## Results

Table 1 suggests that PD participants and control men were similar regarding education level, non-sexual offenses and being a parent, whereas PD participants were less often employed (Cohen’s *d* = −0.90), more often single (*d* = 0.58), had less frequently lived with an intimate partner for 2+ years (*d* = −0.63), had lower IQ (*d* = −0.79)—although on average within the normal range (95–105), and more often self-reported convictions for any sexual offense (*d* = 1.52) or non-contact sexual offenses (*d* = 1.25). Further, PD participants scored higher compared to controls on all dynamic sexual offending risk domains and the predefined composite risk score (Table 2). Effect sizes for risk domains were significant and small (impaired self-regulation, *d* = 0.44), large (sexual preoccupation *d* = 1.11, impaired cognitive empathy and antisocial traits, *d* = 1.18, self-rated risk, *d* = 1.56) and, following inclusion criteria for PD subjects, extremely large (pedophilic attraction, *d* = 2.40). Individual items pertaining to pedophilic attraction and self-rated risk (such as past week watching, socializing with, or sexually interacting with children) were not directly compared across groups, only as part of domain and composite scores. Most notably, however, 12 (22%) PD participants reported prior convictions for sexuality-related crimes; five (9%) stated a contact sex offense against a child (<15 years of age) and eight (15%) any non-contact sexual offense; mostly illegal possession of child sexual abuse material (CSAM), legally referred to as “child pornography”. Eighteen PD subjects (34%) acknowledged *past week*, child-related sexual at-risk behavior, one of them reported actual child sexual interaction during the past week. 66% estimated having a 40% or higher risk of future child-related sexual at-risk behavior; if there existed an easy way to escape detection. Finally, we conducted two *post hoc* sensitivity analyses. First, as IQ is empirically linked to measures of self-regulation and empathy (Vellante et al., 2013; Chien et al., 2017), we correlated group status (PD or control participant) to domain scores with and without adjustment for IQ. Naturally, group status was moderately to strongly significantly correlated with risk scores before adjustment (impaired self-regulation, Spearman’s *r* = −0.22, *p* < 0.05; low empathy and antisocial traits, *r* = −0.55, *p* < 0.01; four-domain risk score, *r* = −0.71, *p* < 0.01). Adjusting for IQ in partial correlation analyses decreased associations somewhat (impaired self-regulation, *r* = −0.13, *p* = 0.19; low empathy and antisocial traits, *r* = −0.48, *p* < 0.01; four-domain risk score, *r* = −0.66, *p* < 0.01). The second *post hoc* sensitivity analysis examined the robustness of the composite score difference by excluding the 12 PD participants with a history of sexuality-related offending. The latter had a composite score Mdn = 8 (*n* = 12, IQR 6.0–9.5) compared to Mdn = 8 in those without (*n* = 41, IQR 6.0–9.0). Exclusion reduced the composite score difference compared to controls from *d* = 2.12 (95% CI: 1.65 to 2.59) to *d* = 2.11 (1.61 to 2.61).

**TABLE 1:** Socio-demographic, criminological and sexuality baseline characteristics among help-seeking, non-correctional adult men with Pedophilic Disorder and matched non-clinical control men in Sweden.

Characteristic	Pedophilic Disorder men (*n* = 55)	Non-clinical control men (*n* = 57)	Cohen’s *d* (95% CI)
*Age, range, M (SD)*	18–66, 36 (12)	18–64, 36 (12)	−0.05 (−0.42 to 0.32)
*Full scale IQ (WAIS-IV), Mdn (IQR)*	101 (23)	115 (15)^a^	−**0.79 (**−**1.17 to**−**0.40)**
*Education, highest level, n (%)*			
Primary school ≤ 9 years	6 (11)	2 (4)	
Secondary school 1–3 years	26 (47)	23 (40)	−0.36 (−0.73 to 0.02)
Postsecondary education	23 (42)	32 (56)	
*Employed, n (%)*	32 (58)	50 (88)	−**0.90 (**−**1.43 to**−**0.38)**
*Parent/guardian, n (%)*	20 (36)	26 (46)	−0.21 (−0.63 to 0.21)
*Currently lives as single, n (%)*	35 (64)	21 (37)	**0.58 (0.16 to 0.99)**
*Ever lived with partner for more than 2 years, n (%)*	26 (47)	42 (74)	−**0.63 (**−**1.06 to**−**0.05)**
*Pedophilic sexual attraction*			
Age of discovery of pedophilic sexual attraction, range, M (SD), Mdn (IQR)^b^	6–39, 18 (7), 16 (13–23)	–	NA
Attraction primarily to boys, *n* (%)	8 (15)	–	NA
Attraction primarily to girls, *n* (%)	43 (78)	–	NA
Attraction to boys and girls, *n* (%)	4 (7)	–	NA
Exclusive attraction to prepubescent children, *n* (%)	12 (22)	–	NA
*Self-reported convictions, n (%)^c^*			
Any sex offense	12 (22)	1 (2)	**1.52 (0.37 to 2.66)**
Contact sex offense (child)^c^	5 (9)	0 (0)	0.96 (−0.24 to 2.16)
Non-contact sex offense (child or adult) or CSAM offense^c,d^	8 (15)	0 (0)	**1.25 (0.09 to 2.42)**
Any non-sexual offense	8 (15)	13 (23)	−0.30 (−0.84 to 0.23)

**Bolded figures are significant at p < 0.05. NA, not applicable. For reasons of completeness regarding Cohen’s d estimates, we exchanged frequencies of “0” to “1” in effect size calculations. ^*a*^Missing data for 1 control. ^*b*^Excluded 4 participants reporting that they “had always known” about their pedophilic sexual attraction and did not provide a specified age. ^*c*^One PD patient reported both contact and non-contact sexual offenses. ^*d*^CSAM offense = possession of child sexual abuse material.**

**TABLE 2:** Comparisons of five domains of dynamic risk factors for child sexual abuse among help-seeking, non-correctional adult men with Pedophilic Disorder and age-matched, non-clinical control men in Sweden.

Risk factor/domain	Pedophilic Disorder men (*n* = 55)	Non-clinical control men (*n* = 57)	Cohen’s *d* (95% CI)
***Pedophilic attraction***			
*Domain risk score* (range 0–3), M (SD)	2.5 (0.6)	0.0 (0.0)	**2.40 (1.91 to 2.89)**
***Sexual preoccupation***			
Sexual Desire Inventory score^a^ (range 12–109), Mdn (IQR)	70 (19)	65 (14)	0.21 (–0.16 to 0.59)
Hypersexual Behavior Inventory score^b^ (range 19–95), Mdn (IQR)	56 (22)	24 (10)	**1.70 (1.26 to 2.14)**
Past week watched child sexual abuse material or observed children for sexual arousal^c^, n (%)			
Not at all	37 (70)	57 (100)	–
A few days	11 (21)	0 (0)	–
More than half the days	1 (2)	0 (0)	–
Nearly every day	4 (8)	0 (0)	–
Past week socializing with children for sexual arousal, n (%)			
Not at all	50 (94)	57 (100)	–
A few days	3 (6)	0 (0)	–
More than half the days/nearly every day	0 (0)	0 (0)	–
Past week direct sexual interaction with children			
Not at all	52 (98)	57 (100)	–
A few days	1 (2)	0 (0)	–
More than half the days/nearly every day	0 (0)	0 (0)	–
*Domain risk score* (range 0–3)^a^, M (SD)	1.6 (0.7)	1.0 (0.2)	**1.11 (0.71 to 1.51)**
***Impaired self-regulation***			
Conners’ Continuous Performance Test aspects most like ADHD norms^b^ (range 0–12), M (SD)	2.3 (1.8)	1.8 (1.5)	0.31 (−0.06 to 0.69)
*Domain risk score* (range 0–3), M (SD)	1.4 (1.0)	1.0 (1.0)	**0.44 (0.06 to 0.81)**
***Impaired cognitive empathy and antisocial traits***			
Ritvo Autism and Asperger Diagnostic Scale – Screening Tool mentalizing subscale score^c^ (range 0–21), Mdn (IQR)	10 (12)	2 (3)	**0.81 (0.43 to 1.20)**
Reading the Mind in the Eyes Test score^c^ (range 0–36), Mdn (IQR)	27 (7)	29 (5)	–0.33 (–0.71 to 0.04)
No. of DSM-5 Antisocial Personality Disorder symptoms (range 0–12), M (SD)	3 (3)	1 (2)	**1.23 (0.83 to 1.64)**
*Domain risk score* (range 0–3)^c^, M (SD)	0.9 (0.8)	0.1 (0.4)	**1.18 (0.78 to 1.59)**
***Self-rated substantial risk of child sexual abuse^b^***, n (%)			
Watching child sexual abuse material or observing children for sexual arousal	35 (66)	0 (0)	–
Socializing with children for sexual arousal	9 (17)	0 (0)	–
Direct sexual interaction with children	11 (21)	0 (0)	–
*Domain risk score* (range 0–3)^b^, M (SD)	1.0 (1.0)	0.0 (0.0)	**1.56 (1.13 to 1.98)**
***Five-domain composite risk score*** (range 0–15)^a,d^, M (SD)	7.5 (1.7)	2.1 (1.1)	**2.12 (1.65 to 2.59)**
***Four-domain composite risk score*** (excluding pedophilic attraction, range 0–12), M (SD)^a^	4.9 (1.9)	2.1 (1.1)	**1.62 (1.18 to 2.05)**

**Bolded figures are significant at p < 0.05. ^*a*^n = 53 and n = 55 subjects for PD and control groups, respectively. ^*b*^n = 53 and 57. ^*c*^n = 54 and 57.*^*d*^For a description of the composite risk score see Supplementary Table 1.*

## Discussion

We compared non-correctional, self-referred adult men diagnosed with DSM-5 Pedophilic Disorder (PD) to age-matched non-clinical control men to elucidate dynamic sexual offending risk factors in a non-forensic clinical context. There were four main findings. First, HBI-assessed hypersexual behavior, lower RAADS-14 mentalizing-based cognitive empathy, and DSM-5 Antisocial Personality Disorder symptoms were much more pronounced among PD cases than controls; effect sizes were large. Second, and contrarily, SDI hyposexuality, ADHD-like self-regulation measured with the CCPT-II test and RMET-based theory of mind impairments did not differ meaningfully between PD and control men. Third, a non-trivial proportion of PD participants reported prior convictions for sexuality-related offending, current child-related sexual at-risk behavior and at least moderate risk of future child-related sexual at-risk behavior. Fourth, when dynamic risk domains were combined into a predefined composite measure, the resulting summary score was very much larger among PD men vs. controls. This large difference remained after excluding the pedophilic attraction domain and each risk domain score was weakly to very much higher in cases compared to controls.

### Socio-Demographic Characteristics

Although education levels were similar, unemployment was moderately more prevalent among PD participants than controls. PD men also had lower IQ, albeit not clinically significantly so. However, both employment rates and higher IQ among controls may partly reflect selection bias toward more cognitively high-functioning individuals volunteering for study participation. In addition, our sensitivity analysis suggested that IQ is unlikely to be a major confounder of the link between group status (case or control) and the composite risk score. Although PD participants had less lifetime experience of living with a partner and were more often single vs. controls, the prevalence of being a parent did not differ.

### Pedophilic Disorder

Since PD was a core inclusion criterion, the lowest possible score in this domain was 2 for PD participants; hence, the effect size for the difference vs. controls on this domain risk score was extremely large. Although reluctance to disclose symptoms cannot be ruled out, null findings regarding PD symptoms among age-matched control men agrees with the notion that persistent pedophilic attraction is infrequent in the general population, perhaps 1% at most (Santtila et al., 2015; Seto, 2018).

### Sexual Preoccupation

SDI baseline scores did not differ meaningfully between PD and control participants, indicating that *hypoactive* sexual motivation is not over-represented in help-seeking, non-forensic PD patients. In contrast, we found a large difference between PD and control men regarding HBI *hypersexualit*y scores, which also contributed substantially to the large difference between PD and control men in the sexual preoccupation domain risk score. The HBI was designed to capture repetitive sexual behaviors in response to dysphoric mood states or stressful life events and the degree to which they are perceived as uncontrollable (Reid et al., 2011). Hypersexual behavior appears to co-occur with paraphilic interest; in fact, paraphilic and hypersexuality comorbidity might fuel help-seeking behaviors (Kafka, 1997; Walton et al., 2017). The median HBI score for our PD subjects is above the suggested cut off-score of 53 when screening for hypersexual disorder (Reid et al., 2011), suggesting such comorbidity and highlighting their motivation to seek help. Simultaneously, the median HBI score in control men (24.0) was lower than that of a large Spanish non-clinical sample (30.5) (Ballester-Arnal et al., 2019).

### Impaired Self-Regulation

We found no significant differences between PD and control men in binary self-regulation measures derived from the CCPT-II, but a moderate, significant difference when integrated into the risk domain score. The CCPT-II is used supportively in both assessment and treatment evaluation of ADHD, but effect sizes of the mean difference between ADHD patients and controls has only been moderate (Hervey et al., 2004). Due to the insufficient discriminative power for ADHD, CCPT-II results are not considered diagnostic. Yet, test-measured executive dysfunction is not a prominent characteristic of our non-forensic PD patients. Importantly, this agrees with studies suggesting that executive dysfunction is more strongly associated with child sexual offending than with PD as such (Joyal et al., 2007; Suchy et al., 2009; Eastvold et al., 2011; Schiffer and Vonlaufen, 2011). Massau et al. (2017) compared men with *pedophilic attraction* with or without prior contact child sexual offending to men *with no pedophilic attraction* but with or without prior contact child sexual offending. One measure of impulsivity was minimally higher in offending compared to non-offending participants (η^2^ = 0.032, *p* = 0.03) but did not differ according to pedophilic attraction.

### Impaired Cognitive Empathy and Antisocial Traits

The RAADS-14 mentalizing subscale, RMET and Antisocial Personality Disorder symptoms all comprised the *impaired cognitive empathy and antisocial traits* domain but contributed differently to the large difference in this domain risk score between PD and control participants.

The RAADS-14 mentalizing subscale attempts to capture Autism Spectrum Disorder (ASD)-related atypical communication and social interaction (Baghdadli et al., 2017) and our PD participants scored considerably higher than controls. Eriksson and colleagues (Eriksson et al., 2013) found a median RAADS-14 mentalizing score of 15 among adult men in Sweden with ASD and 4 among men with other psychiatric disorders than ASD or ADHD. Thus, PD participants’ median fell between those of male ASD patients and men with other psychiatric morbidity, whereas that of our controls agree with those of the non-psychiatric controls (Eriksson et al., 2013). However, importantly, the full diagnostic criteria for ASD were not used by us, and a prior ASD diagnosis did not exclude study participation.

The similarity in RMET scores between cases and controls is somewhat surprising; theoretically, RMET should measure the same construct as the RAADS-14 mentalizing domain. Some research (Oakley et al., 2016) suggests RMET is primarily a measure of *emotion recognition*. Thus, PD participants’ self-rated mentalizing impairments might capture more complex real-life social situations than the RMET. Recently, Schuler et al. (2019) compared men with pedophilic attraction with (*n* = 85) and without (*n* = 72) prior contact child sexual offenses to male non-offending controls (*n* = 128) on several aspects of empathy. The only significant difference between PD and control participants was that those with PD and prior child sexual contact self-reported lower mentalizing ability (*d* = 0.38, *p* < 0.05), and a trend toward impaired emotion attribution (*d* = 0.31, *p* = 0.05) (Schuler et al., 2019). This is partly consistent with the poorer self-rated mentalizing ability and trend toward poorer emotion attribution found among our non-forensic but help-seeking PD men.

The large difference in Antisocial Personality Disorder symptoms should be vetted toward mixed results in prior research. Cohen et al. (2018) studied individuals convicted of child sexual offending (*n* = 50), non-convicted, non-clinical individuals with pedophilic attraction (*n* = 195) and non-PD controls (*n* = 60). Their findings suggested more antisocial traits among convicted offenders, but not among non-convicted PD individuals, compared to controls. Another, likely underpowered study of CSAM offenders (*n* = 23), non-contact offenders (*n* = 15) and contact child molestation offenders (*n* = 49) (Jung et al., 2013) found self-reported antisocial traits to fall within clinically significant ranges across groups. However, more ASPD symptoms in PD participants could partly be inflated by the question specifically asking about “*Repeated illegal acts as an adult*” with previous CSAM offending being somewhat common in our patient population.

### Child-Related Sexually Motivated Behavior: Self-Report and Self-Assessed Risk

The proportion of our PD participants self-reporting any previous conviction of a sexuality-related offense supports the notion that a non-negligible minority of help-seeking PD patients may have prior sexual offense convictions. Additionally, we observed a large proportion of PD participants acknowledging past week, child-related sexual behaviors such as viewing CSAM or observing children for sexual arousal and endorsing a clinically significant risk that they would, if not discovered, watch CSAM or children directly off-line for sexual purposes. First, this supports the notion that watching CSAM may be a diagnostic marker for PD (Seto et al., 2006). Second, it implies that help-seeking individuals with PD may be at risk of committing child sexual abuse and could benefit from risk-reducing treatment. Our findings are consistent with prior research suggesting that most self-identified individuals with PD or respondents in anonymous online surveys endorse past or present CSAM viewing (Riegel, 2004; Neutze et al., 2011; Klein et al., 2015). The present results reflect the *past week* situation when PD patients seek help, and complement the *lifetime* prevalence numbers reported in the seminal German study of 137 self-identified PD individuals where 69% reported previous contact child sexual offending (Neutze et al., 2011).

### Static and Dynamic Risk of Child Sexual Abuse in PD

Static and dynamic risk factors co-occur and interact over time (Craig et al., 2005). To further investigate the overlap of static and dynamic risk among PD patients, future studies might measure static risk factors with tools such as the recently validated Child Pornography Offender Risk Tool (CPORT) (Eke et al., 2019). The CPORT predicts general sexual and child pornography recidivism (AUCs = 0.72–0.74) among individuals adjudicated for CSAM offending but without known contact sexual offenses.

Dynamic measures for sexual offending risk among convicted offenders have been developed (Hanson and Morton-Bourgon, 2009). Two such established measures are the STABLE-2007 and ACUTE-2007, encompassing for example sexual preoccupation, self-regulation problems and antisocial behavior. They both appear to add incremental predictive power beyond static risk alone to risk assessments (Brankley et al., 2019). However, these measures were designed for individuals currently in contact with the legal system for sexual offending, whereas medical professionals generally lack risk measures for non-forensic clinical use. We propose that our dynamic composite risk score might aid in further development of such clinically useful risk assessment and management methods.

### Strengths and Limitations

PD patients were recruited through a national helpline in Sweden, resulting in a consecutive nationwide sample of self-referred, non-forensic participants. Consequently, included PD patients were likely similar to those clinicians would see in sexual medicine and general psychiatry settings, and representative of the target group often addressed in selective prevention of child sexual abuse. Further, the truthfulness of the self-report provided by participants was strengthened by a pseudonymization procedure. Finally, most employed measures had been properly validated in other clinical settings, enabling comparisons of this patient population to those diagnosed with other psychiatric disorders. Regarding limitations, we were not able to prospectively investigate associations between empirically and theoretically suggested dynamic risk measures and actual future CSAM or contact child sexual offending. Further, poor concurrent validity has been suggested across various measures of the cognitive empathy construct (Chen et al., 2017), indicating that it is indeed difficult to measure. Inevitably, any study that requires opting in or help-seeking efforts will be vulnerable to selection biases toward subjects who are indeed motivated to participate. Here by taking part in a project involving a novel treatment addressing impairing sexual preoccupation, impaired cognitive empathy, antisocial traits and self-assessed offending risk. Last, the validity of self-ratings of sexual offending risk and sexually motivated at-risk or abusive behavior against children has not been investigated. However, by safeguarding participant anonymity, these self-reports may be as truthful as possible in a clinical PD study with face-to-face interactions.

Future studies should investigate prospectively the ability of the measures tested here to predict sexual offending, preferably by looking at risk behaviors and situations, self-reports of child sexual offending, and suspicions/arrests for suspected sexual offenses including CSAM crime (Långström et al., 2013). Also, the malleability of these dynamic risk factors or potential treatment effect mediators should be investigated, as attempted in the recently published results of the PRIOTAB RCT of degarelix (Landgren et al., 2020).

## Conclusion

The help-seeking and self-identified PD individuals in this study exhibited offending risk-increasing characteristics. A non-trivial minority also report prior CSAM and direct child sexual offending or current child-related at-risk sexual behavior. This may need consideration in clinical services for help-seeking individuals with PD and supports the idea of PD as a potential treatment target in child sexual abuse prevention. Low-threshold specialist services such as the PrevenTell helpline, the recruitment base for the current study, might be important resources in such prevention efforts. Except from the sexual interest in minors *per se*, risk-related characteristics may include concurrent hypersexuality or sexual preoccupation, mentalizing or cognitive empathy deficits, and antisocial traits. In contrast, non-forensic PD patients may not necessarily differ from healthy general population controls in terms of hypoactive sexual desire and continuous performance tests of inattention, impulsivity, and vigilance. We present a preliminary, dynamic composite risk score that might be useful in developing risk assessment measures for use in child sexual abuse prevention efforts in sexual medicine and general psychiatry.

## Data Availability Statement

The datasets presented in this article are not readily available because the dataset contains sensitive and intimate details about the participants. Requests to access the data will be evaluated on a case by case basis. Such requests should be directed to CR, christoffer.rahm@ki.se.

## Ethics Statement

The studies involving human participants were reviewed and approved by the Swedish Central Ethical Review Board. The patients/participants provided their written informed consent to participate in this study.

## Author Contributions

CR designed the study and collected the data. FW drafted the initial manuscript and CR, VL, and NL reviewed the manuscript for important intellectual content. All authors contributed to data analyses.

## Conflict of Interest

The authors declare that the research was conducted in the absence of any commercial or financial relationships that could be construed as a potential conflict of interest.

## Footnotes

1 www.preventell.se
