# Supplementary material for: Risk Factors for Sexual Offending in Self-Referred Men With Pedophilic Disorder: A Swedish Case-Control Study
Source: Front Psychol. 2020 Nov 26;11:571775. doi: 10.3389/fpsyg.2020.571775 (PMC7726190; doi:10.3389/fpsyg.2020.571775)
Supplement: Supplementary file 1 [file Data_Sheet_1.docx]

SUPPLEMENTAL MATERIAL

Wittström et al. Risk Factors for Sexual Offending in Self-Referred Men with Pedophilic Disorder: A Swedish Case-Control Study. Submitted manuscript.

CONTENTS

1. Description of instruments used in the study
2. Supplemental Table 1. Composite score of dynamic risk for committing child sexual abuse
3. Sexual Child Molestation Risk Assessment (SChiMRA)
4. References

## INSTRUMENTS

#### Sexual Desire Inventory-2 (SDI).

The SDI is a 14-item, self-report measure of frequency and intensity regarding thoughts and feelings about sexual stimuli, both solitary and dyadic (with another person) (1–3). A recent systematic review found the SDI to be the most frequently used sexual desire measure (4). The adapted Swedish version has omitted item 7 from SDI-2, consisting of 13 items with a score range of 12-104 (3). A score <46 is sometimes used clinically as cut-off when screening for hyposexuality. Internal consistency appears high (Cronbach’s alpha .86 and .96 for the dyadic and solitary dimensions, respectively) (3) and the test-retest reliability over a 1-month period is strong (r=.76) (5).

#### Hypersexual Behavior Inventory (HBI).

This is a 19-item scale for self-reported hypersexuality symptoms; excessive and uncontrollable sexual fantasies, urges, and behaviors (6). Hypersexuality is measured along three dimensions; control, consequences, and coping. Respondents rate negative effects of the sexual behavior, if the behavior is uncontrollable, and if it is used to cope with negative emotions (7). An example item is “*My sexual behavior controls my life*” and is responded to on a 5-point Likert scale from 1 (never) to 5 (very often) yielding a total score of 19-95. A score ≥53 points is considered indicative of hypersexuality (6). A systematic review suggested excellent internal consistency (α>.90) and adequate test-retest validity (r>.70) together with good content and construct validity (8).

#### Ritvo Autism and Asperger Diagnostic Scale – Screening Tool (RAADS-14).

This is a 14-item self-report screen for autism spectrum disorder symptoms in adults (9). Items cover mentalizing deficits (seven items), social anxiety (four items) and sensory reactivity (three items). An example item of mentalizing deficits is “*It is hard for me to imagine what others expect of me*”. Items are rated on a 4-point Likert scale; 0 (never true), 1 (true only when I was younger than 16), 2 (true only now) or 3 (true now and when I was young). Hence, higher scores reflect both symptom extent and persistence. The total score for the full questionnaire is 42; 21 (7/14 items) reflect mentalizing deficits (9). RAADS-14 has excellent internal consistency (α>.90) (9). A systematic review of screening and diagnostic tools for ASD found satisfactory psychometric properties and content validity (10), whereas test-retest reliability data have not been published.

#### Conners’ Continuous Performance Test (CCPT-II).

CCPT-II is a performance-based measure of inattention, impulsivity, and vigilance included in a clinical psychologist-led neuropsychological testing procedure (11). It is a 14-minute computerized test where letters are displayed sequentially and responded to, producing T-scores on 12 aspects of the assignment, each scored as 0 (most similar to non-clinical norms) or 1 (most similar to ADHD-norms). Eleven aspects constitute the three domains *inattention* (Omissions, Commissions, Hit RT, Hit RT Std Error, Variability, Detectability (d'), Hit RT ISI Change and Hit RT ISI Change), *impulsivity* (Commissions, Hit RT, Perseverations) and *vigilance* (Hit RT Block Change, Hit SE Block Change). A study of psychology students found acceptable internal consistency (α=.64-.96) and mostly adequate test-retest reliability (r.48-.79 depending on aspect) (12).

#### Reading the Mind in the Eyes Test, Revised Version (RMET).

RMET attempts to objectively measure “theory of mind”; the ability to attribute mental states to another person. The revised version (13) presents 36 pictures depicting an actor’s facial expressions, but revealing only the eyes and the area around them. The respondent selects the expressed mental state from four given alternatives. Subjects have access to a dictionary explaining all alternatives and there is no time limit. The total possible score is 0-36, with higher scores reflecting better emotion attribution. The RMET was administered during neuropsychological testing. Two studies of undergraduate students suggest acceptable internal consistency (Cronbach’s α>.60) and good test-retest reliability (r=.63 and .83) (14,15).

#### Antisocial Personality Disorder symptoms.

Symptoms were assessed with the MINI Neuropsychiatric Interview 6.0: a structured interview covering DSM-IV-TR diagnostic criteria of the most prevalent and important psychiatric disorders (16). Twelve interview items address antisocial behavior and attitudes, coded “yes” (1) or “no” (0). Six items concern the period under age 15 years and another six ≥15 years. We summarized items coded “yes”, yielding a score of 0-12 for each participant. The interview was conducted as part of the psychiatrist-led psychiatric and medical examination and has good test-retest reliability with r>.75 for most included diagnoses. However, agreement with a “gold standard” semi-structured psychiatric interview, varied (17). Reliability and validity information specifically for the antisocial personality disorder module has not been published.

#### Sexual Child Molestation Risk Assessment (SChiMRA).

This is a generic measure, constructed by our research group, addresses self-assessed likelihood of at-risk or sexually abusive behavior against children and the actual self-reported frequency of such at-risk or sexually abusive behavior towards children [<15 years of age] in the past week. Self-reported risk of child sexual offending is tapped by subjects’ responding on a visual analogue scale (0-100%) to: “*How likely is it that you would do any of the following […], if there was an easy way to do it without being caught?* The risk was interpreted as clinically significant if self-rated ≥40%. The three specified behaviors included a) watching CSEM or observing children with sexual intentions; b) socializing with children with sexual intentions; and c) direct sexual interaction with children. The >40% cut-off point chosen for SChiMRA was chosen on the similarity of the VAS-scale used for assessing pain, where patients commonly refer to pain levels above ~40% as moderate rather than mild (18) and has not been validated against actual offending behaviour. Respondents also self-reported *actual at-risk or sexually abusive behavior* against children for the same three behaviors (a-c) on a 4-point Likert scale, where 0 (never), 1 (several days), 2 (more than half of days), 3 (almost every day). Thus, the total scores range from 0 to 9. The rating scale is provided as Supplemental Material.

| Composite score of dynamic risk for committing child sexual abuse^a^ | | | | |
| --- | --- | --- | --- | --- |
| **Risk domain** | **Score definition** | | | |
|  | **0** | **1** | **2** | **3** |
| ***Pedophilic disorder^b^*** | No pedophilic attraction | Pedophilic attraction | Pedophilic attraction + distress or negative consequences | Pedophilic attraction + distress + negative consequences |
| ***Sexual preoccupation^c^*** | Hyposexual according to the SDI | Not hyposexual according to the SDI | Not hyposexual + hypersexual according to the HBI; no ongoing abusive behaviour according to the SChiMRA-B | Not hyposexual + hypersexual + ongoing abusive behaviour |
| ***Impaired self-regulation^d^*** | Normal CCPT II result | 1 abnormal CCPT II domain out of the inattention, impulsivity and vigilance domains | 2 abnormal CCPT II domain out of the inattention, impulsivity and vigilance domains | All 3 abnormal CCPT II domains of inattention, impulsivity and vigilance |
| ***Impaired cognitive empathy and antisocial traits.^e^*** | No abnormality | 1 of RAADS-14 mentalizing domain >10, RMET <22, or current antisocial behaviour according to the MINI | 2 of RAADS-14 mentalizing domain >10, RMET <22, or current antisocial behaviour | RAADS-14 mentalizing domain >10, + RMET <22, + current antisocial behaviour |
| ***Self-rated risk of child sexual abuse^f^*** | Normal SChiMRA-A result | 1 Domain of SChiMRA-A watch, socialize, or interact domain | 2 Domains of SChiMRA-A watch, socialize, or interact domain | 2 SChiMRA-A watch + socialize + interact domain |
| ***Notes:*** SDI, Sexual Desire Inventory; HBI, Hypersexual Behavior Inventory; SChiMRA, Sexual Child Molestation Risk Assessment; CCPT II, Conners´ Continuous Performance Test; RAADS-14, Ritvo Autism and Asperger Diagnostic Scale -14 Screen; RMET, Reading the Mind in the Eyes Test  ^a^ Composite scores ranges from 0 to 15.  ^b^ The three DSM-5 criteria for Pedophilic Disorder; pedophilic attraction, significant distress, and significant negative consequences. | | | | |

## SUPPLEMENTAL TABLE 1.

| ^c^ *Hyposexuality* is defined as a score <45 p on the Sexual Desire Inventory (SDI). *Hypersexuality* is defined as a score ≥53 p on the Hypersexuality Behavior Inventory (HBI). The SChiMRA-B (see Appendix) assesses self-reported frequency of sexually abusive sexually abusive behaviour in the past week (never, several days, more than half of days, or almost every day) regarding watching of, socializing with, and sexual interaction with children, in which occurrence of any sort is scored as significant.  ^d^ Conners’ Continuous Performance Test (CCPT-II) results for inattention, impulsivity and vigilance. Defined as abnormal if at least one aspect within each dimension was most similar (vs. not) to ADHD norms.  ^e^ Self-rated mentalization dimension of the RAADS-14, RMET score and >2 Antisocial Personality Disorder symptoms in adulthood according to the MINI International Neuropsychiatric Interview 6.0.  ^f^ The SChiMRA-A (see Appendix) consists of VAS ratings to the question, “*How likely is it that you would do any of the following if there was an easy way to do it without being detected?”* regarding watching of, socializing with, and sexual interaction with children. A rating of 40% or higher on the VAS was interpreted as a substantial risk. |
| --- |

SChiMRA

Part B

**Think about the last seven days. How often have you engaged in some of the following:**

**1) Watched**

Watched child sexual abuse material, pictures or films, or discreetly observed children/youths for sexual arousal?

Not at all A few days More than half the days Nearly every day

🞏 🞏 🞏 🞏

Comment: _______________________________________________________________

**2) Socialized**

Socialized/talked to/chatted online/texted/sent letters to children/youths for sexual arousal, or in the hopes it may later lead to something more?

Not at all A few days More than half the days Nearly every day

🞏 🞏 🞏 🞏

Comment: _______________________________________________________________

**3) Interacted sexually**

Have physical contact with a child/youth for pleasure or sexual enjoyment, or encourage the child/youth into touching you, or stage other types of more direct sexual/sensual situations remotely (for example through webcam)?

Not at all A few days More than half the days Nearly every day

🞏 🞏 🞏 🞏

Comment: _______________________________________________________________

## REFERENCES

1. King BE, Allgeier ER. The Sexual Desire Inventory as a measure of sexual motivation in college students. Psychol Rep. 2000 Feb;86(1):347–50.

2. Moyano N, Vallejo-Medina P, Sierra JC. Sexual Desire Inventory: Two or three dimensions? J Sex Res. 2017;54(1):105–16.

3. Spector IP, Carey MP, Steinberg L. The Sexual Desire Inventory: Development, factor structure, and evidence of reliability. J Sex Marital Ther. 1996 Sep;22(3):175–90.

4. Cartagena-Ramos D, Fuentealba-Torres M, Rebustini F, Leite ACAB, Alvarenga W de A, Arcêncio RA, et al. Systematic review of the psychometric properties of instruments to measure sexual desire. BMC Med Res Methodol. 2018 19;18(1):109.

5. Fisher TD, Davis CM, Yarber WL. Handbook of Sexuality-Related Measures. Routledge; 2013. 680 p.

6. Reid RC, Garos S, Carpenter BN. Reliability, validity, and psychometric development of the Hypersexual Behavior Inventory in an outpatient sample of men. Sex Addict Compulsivity. 2011 Mar 8;18(1):30–51.

7. Bőthe B, Kovács M, Tóth-Király I, Reid RC, Griffiths MD, Orosz G, et al. The psychometric properties of the Hypersexual Behavior Inventory using a large-scale nonclinical sample. J Sex Res. 2019 Feb;56(2):180–90.

8. Montgomery-Graham S. Conceptualization and assessment of hypersexual disorder: A systematic review of the literature. Sex Med Rev. 2017;5(2):146–62.

9. Eriksson JM, Andersen LM, Bejerot S. RAADS-14 Screen: validity of a screening tool for autism spectrum disorder in an adult psychiatric population. Mol Autism. 2013 Dec 9;4:49.

10. Baghdadli A, Russet F, Mottron L. Measurement properties of screening and diagnostic tools for autism spectrum adults of mean normal intelligence: A systematic review. Eur Psychiatry. 2017;44:104–24.

11. Conners CK. Conners’ Continuous Performance Test II (CPT II V.5). North Tonawanda, NY: Multi-Health Systems, Inc; 2000. 1–16 p.

12. Shaked D, Faulkner LMD, Tolle K, Wendell CR, Waldstein SR, Spencer RJ. Reliability and validity of the Conners’ Continuous Performance Test. Appl Neuropsychol Adult. 2019 Feb 22;1–10.

13. Baron‐Cohen S, Wheelwright S, Hill J, Raste Y, Plumb I. The “Reading the Mind in the Eyes” Test revised version: A study with normal adults, and adults with asperger syndrome or high-functioning autism. J Child Psychol Psychiatry. 2001;42(2):241–51.

14. Fernández-Abascal EG, Cabello R, Fernández-Berrocal P, Baron-Cohen S. Test-retest reliability of the ‘Reading the Mind in the Eyes’ test: a one-year follow-up study. Mol Autism. 2013 Sep 11;4(1):33.

15. Vellante M, Baron-Cohen S, Melis M, Marrone M, Petretto DR, Masala C, et al. The “Reading the Mind in the Eyes” test: systematic review of psychometric properties and a validation study in Italy. Cognit Neuropsychiatry. 2013 Jul;18(4):326–54.

16. Sheehan DV, Lecrubier Y, Sheehan KH, Amorim P, Janavs J, Weiller E, et al. The Mini-International Neuropsychiatric Interview (M.I.N.I.): the development and validation of a structured diagnostic psychiatric interview for DSM-IV and ICD-10. J Clin Psychiatry. 1998;59 Suppl 20:22-33;quiz 34-57.

17. Sheehan D, Lecrubier Y, Harnett Sheehan K, Janavs J, Weiller E, Keskiner A, et al. The validity of the Mini International Neuropsychiatric Interview (MINI) according to the SCID-P and its reliability. Eur Psychiatry. 1997 Jan 1;12(5):232–41.

18. Jensen MP, Chen C, Brugger AM. Interpretation of visual analog scale ratings and change scores: a reanalysis of two clinical trials of postoperative pain. J Pain Off J Am Pain Soc. 2003 Sep;4(7):407–14.
